# Methicillin-Sensitive Staphylococcus aureus Prostatic Abscess in a Diabetic Patient: Successful Management With Transurethral Resection and Review of the Literature

**DOI:** 10.7759/cureus.96904

**Published:** 2025-11-15

**Authors:** Yuki Matsui, Satoshi Asakura, Wahei Yanagida, Hirotaka Kishi, Takashi Fukagai

**Affiliations:** 1 Department of Urology, Showa Medical University School of Medicine, Tokyo, JPN

**Keywords:** diabetes mellitus, prostatic abscess, staphylococcus aureus, surgical drainage, turp

## Abstract

Prostatic abscess (PA) is a rare but potentially life-threatening complication of acute bacterial prostatitis, most often caused by Gram-negative organisms such as *Escherichia coli*. *Staphylococcus aureus*, although uncommon, has emerged as a clinically significant pathogen associated with aggressive disease courses and higher rates of surgical intervention. We report the case of a 40-year-old man with poorly controlled type 2 diabetes mellitus who presented with persistent fever, perineal pain, and urinary symptoms for two weeks despite empirical antibiotic therapy with intravenous meropenem (1 g every 8 hours). Laboratory evaluation showed leukocytosis and markedly elevated C-reactive protein. Imaging with contrast-enhanced computed tomography revealed a large multiloculated PA measuring 3.0 × 4.0 cm. Blood and abscess cultures both grew methicillin-susceptible *S. aureus*. The patient underwent transurethral resection of the prostate with drainage, followed by intravenous cefazolin for 14 days, achieving complete clinical recovery and normalization of inflammatory markers at six months. This case highlights the importance of considering *S. aureus* as a causative pathogen in PA, particularly in diabetic or immunocompromised patients with refractory symptoms. Early imaging, microbiological confirmation, and individualized management, including surgical drainage for large or multiloculated abscesses, are crucial for favorable outcomes.

## Introduction

Prostatic abscess (PA) is an uncommon but potentially life-threatening sequela of acute bacterial prostatitis, accounting for approximately 0.2% to 2.5% of prostatitis cases [1]. The condition typically arises in older men or those with underlying comorbidities, particularly diabetes mellitus, chronic kidney disease, or immunosuppression [2]. Historically, PA has been predominantly caused by Gram-negative enteric bacteria such as *Escherichia coli* and *Klebsiella pneumoniae* [3]. However, recent studies have highlighted a growing number of Gram-positive infections, most notably *Staphylococcus aureus* (SA), which can result in more aggressive disease courses and higher rates of systemic dissemination [4,5].

Although PA is a recognized urologic emergency, *Staphylococcus aureus* prostatic abscess (SA-PA) remains a rare clinical entity, with fewer than 50 cases documented in the literature, and methicillin-sensitive strains (MSSA) comprising a minority of these reports [6]. Due to its rarity, standardized diagnostic and management protocols for SA-PA are lacking. The clinical presentation is often nonspecific, necessitating high clinical suspicion, and optimal management frequently requires a combination of targeted antibiotic therapy and surgical drainage. Imaging modalities, particularly contrast-enhanced computed tomography (CT), play a central role in diagnosis and in guiding the choice of drainage method by delineating the size, location, and extent of the abscess cavity. CT helps distinguish unilocular from multiloculated collections and defines whether the abscess is confined to the prostate or extends beyond the capsule, thereby aiding in selecting between transurethral and percutaneous approaches [3,7]. Here, we report a case of MSSA-induced PA in a patient with poorly controlled diabetes mellitus that was successfully managed with transurethral resection of the prostate (TURP). We also review the relevant literature to highlight diagnostic challenges, pathogen-specific features, and optimal treatment strategies for this uncommon but clinically important condition.

## Case presentation

A 47-year-old male with a two-year history of untreated type 2 diabetes mellitus presented to the emergency department with a one-week history of high-grade fever (maximum 39.2°C), progressive fatigue, myalgia, and decreased oral intake. He also reported dysuria, urinary frequency, and hesitancy that had worsened over the preceding three days, along with perineal discomfort and erythema of the lower extremities.

Medical history

The patient had been diagnosed with type 2 diabetes mellitus during a routine health screening two years earlier, but had declined treatment and lifestyle modifications. He had no history of urological procedures, catheterization, or urinary tract infections. He denied recent travel, sexual risk factors, or immunosuppressive medication use.

Physical examination

On arrival, the patient appeared acutely ill with fever (38.8°C), tachycardia (102 bpm), and mild hypotension (95/60 mmHg). Bilateral lower extremity erythema and warmth consistent with cellulitis were observed. Digital rectal examination revealed an enlarged, markedly tender prostate with fluctuant areas, highly suggestive of abscess formation.

Laboratory investigations

Laboratory findings are summarized in Table 1. The results revealed marked leukocytosis with neutrophilia and markedly elevated C-reactive protein (CRP), consistent with acute bacterial infection. Severe hyperglycemia and elevated HbA1c confirmed poorly controlled diabetes mellitus. Blood and urine cultures both grew MSSA, confirming the causative pathogen.

**Table 1:** Laboratory investigations Laboratory data on admission demonstrating systemic inflammation, severe hyperglycemia, and methicillin-susceptible Staphylococcus aureus (MSSA) bacteriuria and bacteremia, consistent with prostatic abscess secondary to poorly controlled diabetes mellitus.

Laboratory investigations			
Parameter	Result (on admission)	Reference range	Interpretation
White blood cell count	16,400 /μL	3,500–9,000 /μL	↑ Elevated
Neutrophils	85%	40–70%	↑ Elevated
C-reactive protein (CRP)	22.3 mg/dL	<0.3 mg/dL	↑ Markedly elevated
Serum glucose	456 mg/dL	70–140 mg/dL	↑ Severe hyperglycemia
HbA1c	14.00%	4.6–6.2%	↑ Poor glycemic control
Urinalysis (WBC)	>50 /HPF	<5 /HPF	↑ Elevated
Blood culture	*Staphylococcus aureus* (MSSA)	Negative	Positive
Urine culture	*Staphylococcus aureus *(MSSA)	Negative	Positive

**Figure 1:**
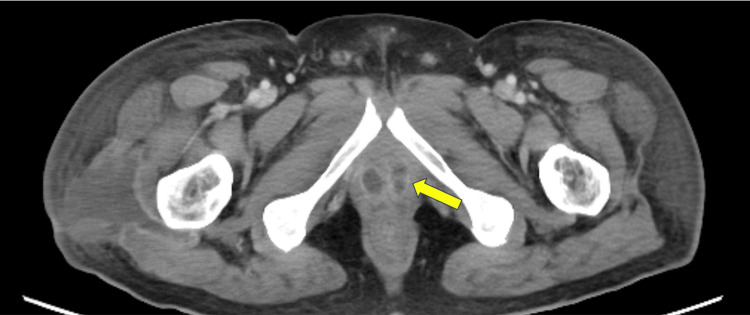
CT Imaging of a Multiloculated Prostatic Abscess CT shows a multiloculated prostatic abscess (arrow) with rim enhancement and central hypodensity, measuring approximately 4.2 × 3.8 cm.

Imaging studies: Contrast-enhanced computed tomography (CT) of the pelvis revealed a multiloculated PA measuring 4.2 × 3.8 cm with rim enhancement and central hypodensity (Figure 1). The lesion involved the transition zone and compressed the bladder neck but showed no extraprostatic extension.

**Figure 2:**
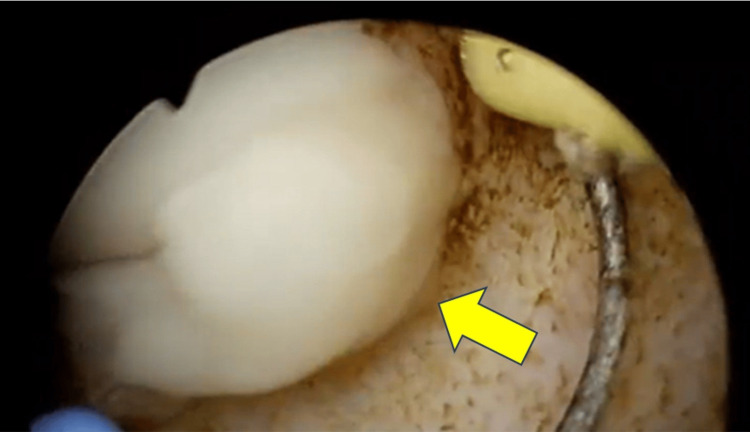
Intraoperative Endoscopic View of Prostatic Abscess Drainage Intraoperative endoscopic view during TURP, showing drainage of thick purulent material from the abscess cavity. TURP: transurethral resection of the prostate

Treatment and clinical course: Given the abscess size, multiloculated pattern, and systemic toxicity, surgical drainage was performed. The patient underwent TURP under general anesthesia, with drainage of approximately 40 mL of pus (Figure 2). Abscess fluid culture confirmed MSSA sensitive to cefazolin and vancomycin. Postoperatively, intravenous cefazolin (2 g every 8 hours) was administered for 14 days, along with insulin therapy for glycemic control. Clinical condition improved within 48 hours, with resolution of fever and normalization of inflammatory markers by day 5.

Outcome

Urinary symptoms resolved, and the patient achieved good glycemic control with newly initiated diabetes management. At six-month follow-up, he remained asymptomatic with no recurrence (HbA1c: 7.2%).

## Discussion

Clinical significance and epidemiology

PA remains an uncommon but clinically significant entity [1-3]. Its diagnosis and management in the post-antibiotic era have been described previously [2]. Although its incidence has declined in the antibiotic era, delayed diagnosis can still result in sepsis, fistula formation, such as rectourethral or perineal fistulae, or even mortality. Our case emphasizes the importance of maintaining a high index of suspicion in high-risk patients, such as those with poorly controlled diabetes, where urinary and systemic symptoms may overlap.

Pathogen-specific differences

*Staphylococcus aureus*-associated PA demonstrates distinct clinical features compared to Gram-negative organisms such as *Escherichia coli*. Reported cases involve younger patients, a more aggressive clinical course, and higher rates of drainage [6]. These features likely reflect the strong tissue-destructive capacity of *S. aureus*, predisposing to multiloculated abscesses and antibiotic failure. By contrast, *E. coli*-induced abscesses more often respond to conservative therapy [7]. A summary of previously reported cases of *S. aureus* PA, including causative organism, treatment modality, and outcomes, is presented in Table 2.

**Table 2:** Reported cases of Staphylococcus aureus prostatic abscess in the literature Summary of representative reports of *Staphylococcus aureus* prostatic abscess, including causative organism, treatment modality, and clinical outcome. TURP: transurethral resection of the prostate; MRSA: methicillin-resistant *Staphylococcus aureus*; MSSA: methicillin-susceptible *Staphylococcus aureus*

Author (Year)	Pathogen Type	Number of Cases	Treatment Method	Outcome	Notes
Carroll et al. (2017)	MRSA/MSSA	42	TRUS drainage/TURP/antibiotics	Most recovered	Literature review
Deshpande et al. (2013)	MRSA	1	TRUS drainage + antibiotics	Recovered	Community-acquired MRSA
Vyas and Endy (2020)	MRSA	1	Percutaneous drainage + antibiotics	Recovered	Associated renal abscess
Oshinomi et al. (2018)	Mixed (Gram–/+)	18	TURP or TRUS-guided drainage	Recovered	Large institutional series
Present case (2025)	MSSA	1	TURP + antibiotics	Recovered	Poorly controlled diabetes

Differential diagnosis and diagnostic workup

The differential diagnosis of PA includes acute bacterial prostatitis, urinary tract infections, and renal stone disease. Clinical overlap of urinary and systemic symptoms may delay diagnosis, emphasizing the need for a structured diagnostic approach. Laboratory investigations, including PSA, blood and urine cultures, and abscess fluid cultures, are essential for confirming the diagnosis and guiding targeted antimicrobial therapy. Elevated PSA levels may reflect inflammation rather than malignancy and should be interpreted in the clinical context [2-3].

Importance of imaging and classification

Contrast-enhanced CT is central to both diagnosis and treatment planning. Abscess morphology - focal, diffuse, or multiloculated - offers a practical framework for decision-making. Previous reports have shown that abscesses larger than 2 cm or multiloculated are more likely to fail conservative therapy [6-7]. High-resolution pelvic CT not only aids in initial diagnosis but also provides valuable prognostic information by monitoring abscess resolution during follow-up. Imaging-based classification is therefore crucial for risk stratification and guiding timely intervention [1,6].

Role of comorbidities

Diabetes mellitus is the most frequently reported comorbidity and impairs host defenses through defective neutrophil function [6]. This predisposition is particularly relevant for *S. aureus*, whose virulence factors promote abscess formation. Other reported risk factors include chronic liver disease, indwelling catheters, and immunosuppression, but diabetes remains the most consistent [6]. Optimizing metabolic control should therefore be considered an integral part of management.

Treatment considerations

Antibiotics are essential, but their efficacy depends on both pathogen and morphology. *S. aureus*, especially methicillin-resistant *Staphylococcus aureus* (MRSA), may require glycopeptides or beta-lactams with targeted activity. In large or multiloculated abscesses, surgical drainage is usually necessary [6]. TURP offers direct access and thorough evacuation, and in our case, resulted in rapid clinical improvement without recurrence [7]. Early surgical intervention should be strongly considered in patients with systemic toxicity or inadequate response to antibiotics.

Monitoring and outcomes

Serial inflammatory markers such as WBC and CRP can guide treatment response. Persistently elevated values should prompt reevaluation or repeat drainage. An untreated PA can lead to severe sepsis, fistula formation, and mortality. Postoperative complications, including urethral strictures or bleeding, may occur following surgical drainage, highlighting the importance of careful perioperative management. Risk of recurrence appears higher with incomplete evacuation and uncontrolled comorbidities, underscoring the importance of follow-up for at least three to six months, including clinical assessment and imaging when indicated. In our case, no recurrence was observed during six months of follow-up. Reported recurrence rates of PA, however, range from 6% to 10%, particularly in cases with incomplete drainage or poorly controlled diabetes [2,3].

## Conclusions

This case emphasizes the clinical significance of recognizing *S. aureus* as a potential causative pathogen of PA, particularly in individuals with underlying metabolic or immunocompromised conditions. Timely diagnostic imaging and early intervention are pivotal in reducing morbidity and preventing systemic dissemination. Although conservative management with antibiotics alone may be effective in small, uniloculated, and clinically stable cases reported in the literature, our case highlights that surgical drainage via transurethral resection remains the preferred approach for multiloculated or refractory abscesses. The present report highlights the importance of a tailored, evidence-based approach integrating microbiological identification, imaging findings, and patient-specific factors to achieve optimal clinical outcomes.
